# Study of Morphological, Structural, and Strength Properties of Model Prototypes of New Generation TRISO Fuels

**DOI:** 10.3390/ma15144741

**Published:** 2022-07-06

**Authors:** Inesh Kenzhina, Petr Blynskiy, Artem Kozlovskiy, Meiram Begentayev, Saulet Askerbekov, Zhanna Zaurbekova, Aktolkyn Tolenova

**Affiliations:** 1 Laboratory of Structural Materials for Nuclear Power Plants, Al-Farabi Kazakh National University, Almaty 050032, Kazakhstan; blynskiy89@mail.ru (P.B.); saulet@list.ru (S.A.); zzha@mail.ru (Z.Z.); aktolkyntolen@gmail.com (A.T.); 2 Advanced Electronics Development Laboratory, Kazakh-British Technical University, 59 Tole bi St., Almaty 050000, Kazakhstan; 3 Laboratory of Solid State Physics, The Institute of Nuclear Physics, Almaty 050032, Kazakhstan; kozlovskiy.a@inp.kz; 4 Department of General Physics, Satbayev University, Almaty 050032, Kazakhstan; mbegentaev@gmail.com

**Keywords:** TRISO fuel, nuclear fuel, mechanical testing, nuclear power, structure

## Abstract

The purpose of this work is to characterize the morphological, structural, and strength properties of model prototypes of new-generation TRi-structural ISOtropic particle fuel (TRISO) designed for Generation IV high-temperature gas reactors (HTGR-type). The choice of model structures consisting of inner pyrolytic carbon (I-PyC), silicon carbide (SiC), and outer pyrolytic carbon (O-PyC) as objects of research is motivated by their potential use in creating a new generation of fuel for high-temperature nuclear reactors. To fully assess their full functional value, it is necessary to understand the mechanisms of resistance to external influences, including mechanical, as in the process of operation there may be external factors associated with deformation and leading to the destruction of the surface of fuel structures, which will critically affect the service life. The objective of these studies is to obtain new data on the fuel properties, as well as their resistance to external influences arising from mechanical friction. Such studies are necessary for further tests of this fuel on corrosion and irradiation resistance, as closely as possible to real conditions in the reactor. The research revealed that the study samples have a high degree of resistance to external mechanical influences, due to the high strength of the upper layer consisting of pyrolytic carbon. The presented results of the radiation resistance of TRISO fuel testify to the high resistance of the near-surface layer to high-dose irradiation.

## 1. Introduction

This study is motivated by the increase in energy consumption and the efforts underway to search for alternative energy sources, including active studies in the field of nuclear and thermonuclear energy. In Kazakhstan, in recent years, new materials for future nuclear and nuclear fuel cycle facilities have been widely studied [1,2,3,4]; reactor [5,6,7,8,9,10,11,12,13,14,15,16] and out-of-pile [17,18,19] experiments simulating their operating conditions have been conducted. One area of such work is corrosion testing of materials for the high-temperature gas reactor (HTGR), a Generation IV in the early design stage [20,21,22,23]. The concept of Generation IV reactors is to improve safety, reliability, and cost-effectiveness, which will allow them to compete more effectively with conventional power generation methods. One of the promising trends in the design of Generation IV reactors is the creation of high-temperature gas-cooled reactors, whose main purpose is not only to produce technological heat, but also to produce hydrogen [24,25,26]. According to the development concept and roadmap for Generation IV power systems, the main questions to be answered before commissioning high-temperature gas-cooled reactors are to determine their stability, safety, reliability, and physical protection against radiation damage [27,28,29,30].

One way to improve the safety and reliability of new-generation nuclear reactors is to use different types of nuclear fuel [31,32,33]. One of these types of fuel is tri-structural isotropic nuclear fuel (TRISO or TRi-structural ISOtropic), which is small UO_2_, UO_x_C_y_, or UC_z_ particles coated with layers of pyrocarbon and silicon carbide [34,35,36]. The use of such coatings allows for several levels of safety, as well as the ability to withstand high-temperature loads and high internal gas pressures without releasing uranium nuclear fission products into the environment [37,38]. As is known, the use of silicon carbide coatings as protection against external influences, including mechanical impacts, is widespread in various fields of science and technology. However, the use of silicon carbide coatings in nuclear power as coatings of nuclear fuel requires additional research and experiments [39,40,41,42,43,44,45]. This is due primarily to the fact that any modification of one of the TRISO fuel layers entails changes in the entire fuel structure, therefore requiring further research [46,47].

It is also worth noting that an important area of research in the field of new fuels is the study of the radiation resistance of fuel elements to radiation damage and the kinetics of resistance to radiation-induced processes accompanied by embrittlement or destruction of the damaged material. The most dangerous among radiation damage is neutron radiation, which can lead to the formation of radiation defects, the consequence of which is neutron embrittlement and partial destruction due to swelling processes. The interest in these studies is due to the need to obtain data on the change in the properties of fuel materials during the accumulation of radiation damage and to determine their resistance to radiation, which will identify opportunities in the potential use of these materials. Today, there are a number of scientific studies related to the study of radiation defects in ceramics, structural materials, and new types of inert matrices of nuclear fuel, and the interest in these studies increases every year [48,49,50,51,52,53,54]. 

Based on the above, the main goal of this work is to study the morphological, structural, and strength properties of model prototypes of a new generation TRISO fuel consisting of several layers of pyrolytic carbon and silicon carbide. Obtaining data on the properties of this fuel prototype will allow the development of further strategies for corrosion and reactor testing, which require a large amount of a priori data on the initial samples and their characteristics. The paper also presents data on the resistance of selected objects of study to radiation damage caused by irradiation with heavy ions: He^2+^ (40 keV) with fluences of 10^16^–10^17^ ions/cm^2^ and high-energy Kr^15+^ (150 MeV) and Xe^22+^ ions (220 MeV) with fluences of 10^13^–10^15^ ions/cm^2^ at temperatures of 1000 K.

## 2. Research Methods

The test samples of TRISO fuel prototypes were obtained for further research from Nuclear Fuel Industries, Ltd. (Muramatsu, Tokai-mura, Japan), which is developing this type of fuel for testing. The work [55] presents a detailed description of the technology for manufacturing such samples, which were taken as objects of this study. The objects of study in this work were models of the TRISO fuel prototype, in which the uranium oxide core was replaced by silicon carbide. All other coatings were applied in full accordance with the manufacturing technology of the original fuel prototype [55]. The purpose of manufacturing such mock-ups of the fuel prototype is to expand the range of laboratories for independent testing of its wear resistance and anti-corrosion properties, while observing the principles of non-proliferation of nuclear materials and radiation safety. The manufacturing technology of such a prototype cannot yet be disclosed for reasons of intellectual property protection.

Replacing the core of uranium oxide with silicon carbide is justified in the study of the strength and corrosion properties of the outer protective layers of the test sample, since it does not affect the properties of the coating material because silicon carbide surpasses uranium oxide in mechanical properties. The results obtained in the study of wear resistance are quite interesting and can be exemplary when conducting full tests of the original fuel.

The TRISO fuel prototypes with a silicon carbide fuel core with different layers were chosen as the objects of the study. The choice of silicon carbide as the core of fuel particles is due to its high resistance to heating, as well as radiation resistance. As a rule, for model objects, either silicon carbide or zirconium dioxide is used as the core material. The choice of these prototypes of nuclear fuel as objects of study, without a core filled with uranium or plutonium fuel, is due to the need to conduct test experiments aimed at studying the stability of the outer shells to mechanical influences. The main technical characteristics of the prototypes under study are presented in Table 1.

The study of morphological features, as well as the elemental composition of the studied samples, was carried out using methods of scanning electron microscopy (Hitachi TM4000 microscope, Hitachi, Tokyo, Japan) and atomic force microscopy (AIST-NT SPM microscope, AIST-NT, Moscow, Russia).

The structural characteristics and phase composition were determined using X-ray diffraction performed on a D8 Advance diffractometer (Bruker, Karlsruhe, Germany). (Imaging parameters: Cu-kλ = 1.54 Å, 2θ = 10–80°, step 0.01°).

The strength characteristics were determined using the method of wear resistance tests at different loads (100–500 N) and determining the coefficient of dry friction. Tests were carried out on a series of samples (10 pieces) using the standard methodology of GOST [56]. Tests with different loads make it possible to assess the degree of resistance of the material to various pressures that occur during operation. For studies on resistance to mechanical stress, the samples were placed on special holders, then half-filled with epoxy resin in order to avoid displacement of the samples during external action: indentation or determination of the dry friction coefficient.

Determination of the resistance of the TRISO surface layer to radiation damage associated with their accumulation during irradiation was performed by irradiating the investigated objects with low-energy He^2+^ ions (40 keV) with fluences of 10^16^–10^17^ ions/cm^2^ and high-energy Kr^15+^ (150 MeV) and Xe^22+^ ions (220 MeV) with fluences of 10^13^–10^15^ ions/cm^2^ at temperatures of 1000 K. Irradiation was carried out at the heavy ion accelerator DC-60 (Nur-Sultan, Kazakhstan), located on the grounds of the Institute of Nuclear Physics. The choice of ions and irradiation conditions was determined by the simulation of radiation damage comparable to that in high-temperature nuclear reactors. Determination of the irradiation effect was evaluated by the resistance to cracking under single compression of samples and determination of the hardness value when indenting samples before and after irradiation. For irradiation, the special holders for the samples were capable of heating, making it possible to carry out direct temperature heating of the samples during irradiation.

## 3. Results and Discussion

**Figure 1:**
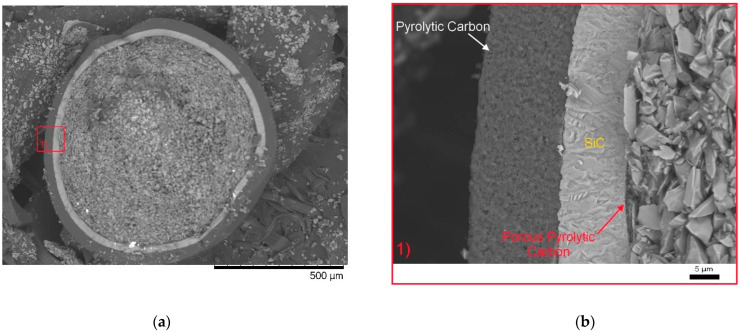
SEM image of the internal structure of the TRISO sphere: ((**a**) side chip; (**b**) detailed image of ceramic layers (1) enlarged image of the side cleavage area).

Spherical particles with a diameter of 1 mm, representing model particles of fuel TRISO, were the objects of the research. During the experiments, it was determined that the initial samples were spheres consisting of three different layers and a core (Figure 1).

**Figure 2:**
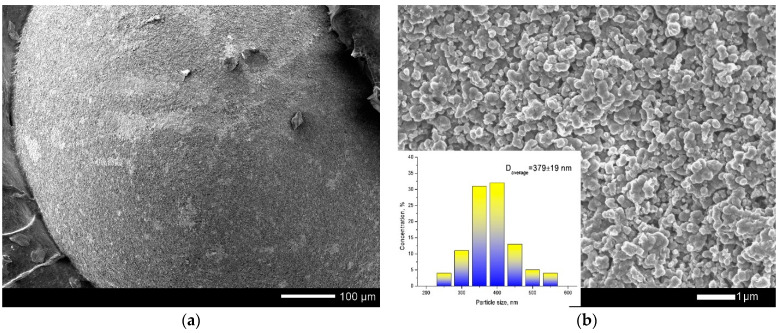
(**a**) SEM images of TRISO sphere surface; (**b**) detailed image of TRISO sphere surface and grain size diagram.

According to the data obtained, the structure of the studied particles consists of the upper layer of pyrolytic carbon, the second layer of silicon carbide, a thin layer of porous pyrolytic carbon, and a core of silicon carbide and carbon particles. The first pyrolytic carbon layer is a densely packed structure consisting of grains with an average size of 300–400 nm (Figure 2).

**Figure 3:**
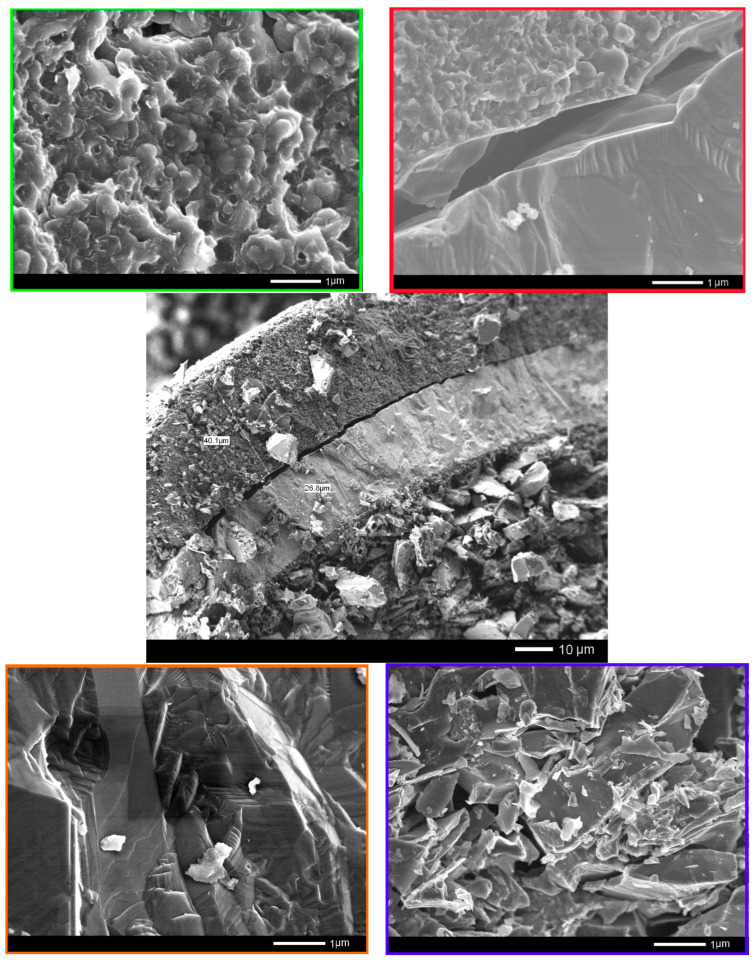
SEM images of the internal structure of the TRISO sphere.

Figure 3 shows detailed SEM images of the layer boundaries and their internal structures.

**Figure 4:**
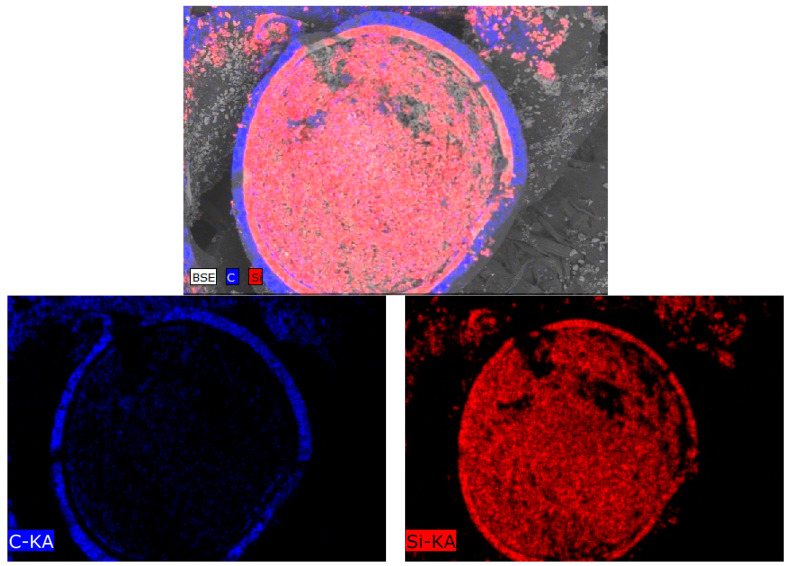
Results of TRISO sphere internal structure mapping.

**Figure 5:**
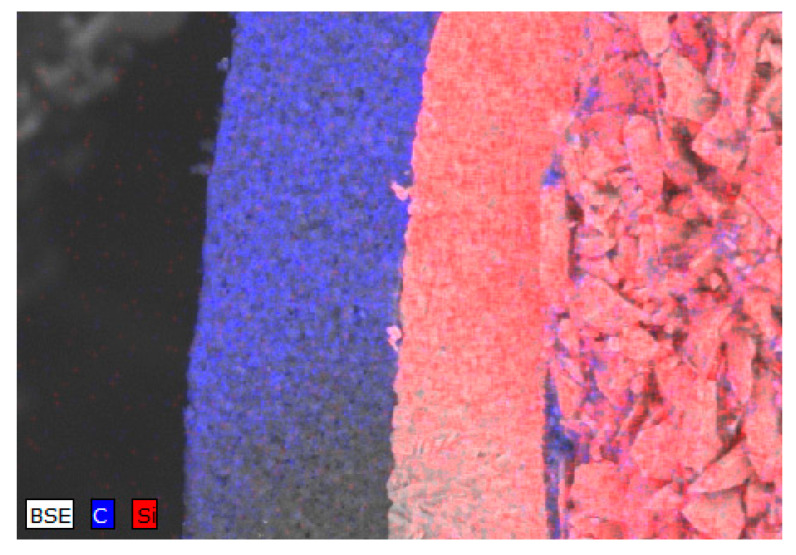

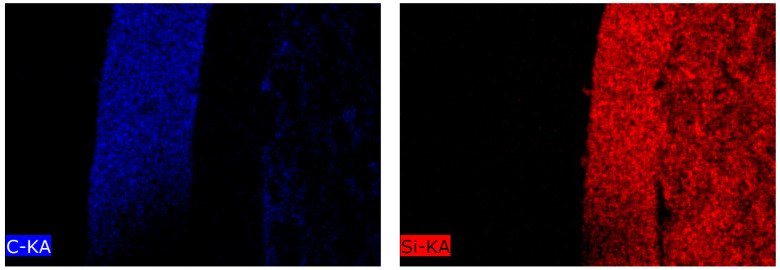
Results of TRISO layer structure mapping.

From the SEM images presented in Figure 3, we can see that the internal structures of the layers are significantly different, as well as having clear boundaries separating them from each other. The thickness of the first layer is 40–42 µm. The data of energy dispersive analysis of the first layer confirms the fact that the first layer consists entirely of pyrolytic carbon with a graphite-like structure (see data of the mapping results presented in Figure 4 and Figure 5). At the same time, the inner structure of the layer is a porous structure consisting of spherical grains. The estimate of the porosity value for this layer is 5–5.5%.

The second layer, which is 26–27 µm thick, is a ceramic coating based on silicon carbide. The inner core is a mixture of large grains ranging in size from 1 to 3 μm. An amorphous layer of pyrolytic carbon with a thickness of 0.5–1 µm is observed between the core and the ceramic layer of silicon carbide.

According to elemental analysis, the inner part of the core is filled with silicon carbide particles with a small amount of silicon oxide impurities (no more than 3–5%).

**Figure 6:**
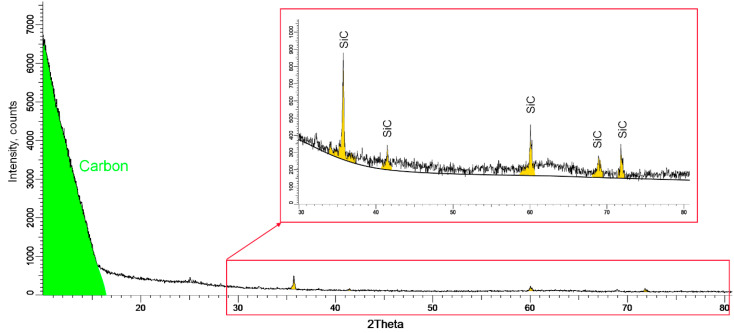
X-ray diffractogram of the investigated TRISO sample.

To determine the structural characteristics and phase composition, as well as the porosity of the studied samples, the method of X-ray diffraction was applied. The general view of the X-ray diffractogram shown in Figure 6 indicates the presence of two phases in the structure of the samples under study. The main phase, characterized by a halo peak in the region of 2θ = 10–17°, corresponds to the structure of amorphous pyrolytic carbon (PyC). Low-intensity X-ray reflections at 2θ = 36°, 41°, 60.5°, 68.7°, and 72.8° correspond to the silicon carbide (SiC) phase with a cubic crystal lattice type and F-43m(216) spatial syngony. Applying the Rietveld full-profile method to evaluate the contributions of the two phases in the structure of the investigated samples, it was found that the phase ratio is PyC:SiC = 73.3:26.7. Analysis of the crystal lattice parameters and volume for the SiC phase made it possible to establish that the integral porosity value of the examined samples is 1.61%, at a phase density of 3.134 g/cm^3^. The degree of crystallinity of the ceramic layer of silicon carbide is more than 95%, which indicates a high degree of structural ordering.

**Figure 7:**
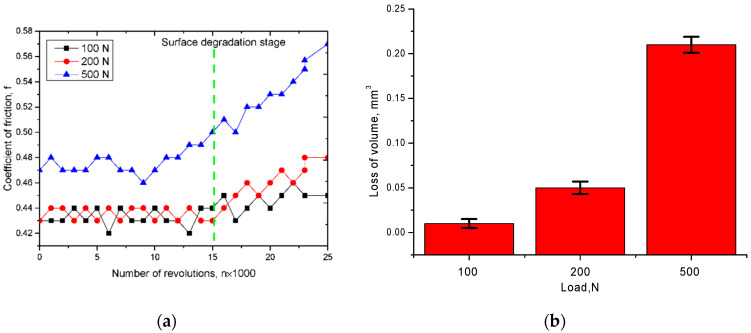
(**a**) Diagram of the change in the dry friction coefficient depending on the load force; (**b**) diagram of the change in the amount of wear during testing.

The study of strength characteristics was carried out by determining the coefficient of dry friction of the samples under different loads on the sample in order to determine the strength of the sphere surface. Wear resistance tests were carried out by rolling with 10% slip. The values of the applied loads were 100, 200, and 500 N. The test results are shown in Figure 7. The presence of small changes in the values of the coefficient of dry friction is due to the emergence of small micro-fractures or micro-cracks arising in the friction process, which create small obstacles in the friction process, which leads to fluctuations in the value of the coefficient.

As can be seen from the presented data, in the case of loads 100–200 N the value of the dry friction coefficient is 0.43–0.44 and is kept in this range for a sufficient number of test cycles (more than 15,000 cycles). After 15,000 cycles of consecutive rolling, there is a slight increase in the dry friction coefficient, which indicates the beginning of degradation and partial destruction of the particle surface, leading to an increase in friction. However, these changes do not exceed 1–5%, which indicates high resistance to wear resistance. When the load is increased up to 500 N, an increase in the dry friction coefficient up to 0.45–0.46 is observed, which indicates greater slip resistance of the particles at higher loads. Meanwhile, the increase in the dry friction coefficient during successive rolls shows an earlier increase in the coefficient and a greater loss of volume as a result of long-term tests than at 100 and 200 N loads.

**Figure 8:**
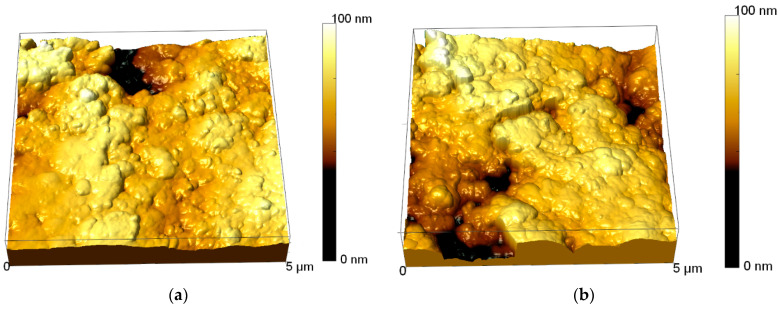
AFM images of TRISO sphere surface before and after friction tests: (**a**) initial sample; (**b**) after friction tests at 500 N.

Figure 8 shows AFM images of the surface of the studied particles before and after the tests at a load of 500 N. The obtained data show that in the initial state the sphere surface has a developed surface consisting of fine-grained inclusions with a high degree of uniformity. The average value of surface roughness is 51 ± 5 nm. For samples after wear resistance tests, there is a sharp change in surface topography with the formation of irregularities and crater formations, indicating partial surface degradation. In this case, the value of the average roughness for the samples after the tests increases more than 2.5 times and is 132 ± 7 nm.

The data obtained indicate a high degree of resistance of the studied specimens to wear resistance tests under low loads over a long period of time.

**Figure 9:**
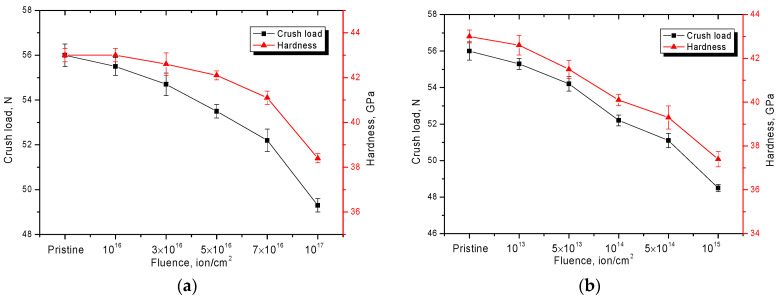

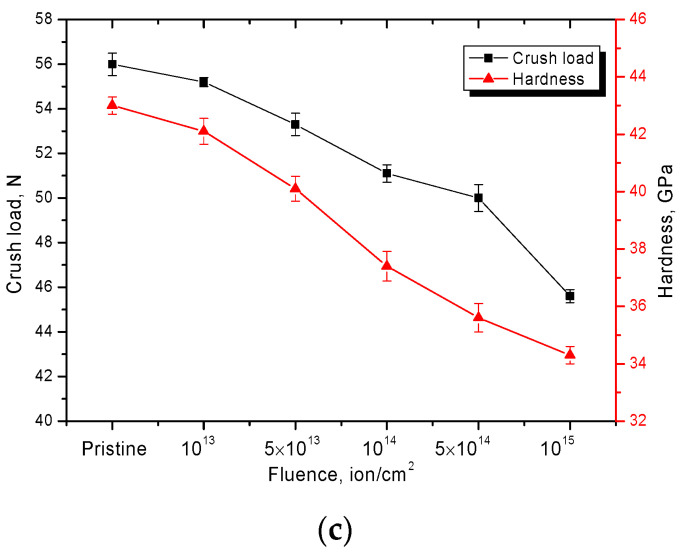
Results of changes in the strength properties of TRISO samples to irradiation: (**a**) irradiation with He^2+^ ions; (**b**) irradiation with Kr^15+^ ions; (**c**) irradiation with Xe^22+^ ions.

Figure 9 shows the results of measurements of the fracture toughness values of the investigated samples subjected to irradiation with different types of ions and irradiation fluences. The choice of He^2+^ ions for irradiation is due to the possibility of modeling the processes of helium swelling during irradiation, as well as the formation of gas-filled bubbles during implantation of He^2+^ ions with the subsequent formation of He-V type complexes, which can agglomerate. At the same time, the swelling effects are most interesting in the near-surface layer, where degradation can lead to accelerated destruction and decrease in the particles’ resistance to cracking and accelerated fracture under mechanical stresses. The choice of Kr^15+^ (150 MeV) and Xe^22+^ ions (220 MeV) allows for the simulation of radiation damage caused by uranium fission fragments in nuclear fuel. The fluence ranges were chosen to establish the dynamics of damage accumulation and their effect on strength properties.

As can be seen from the presented data for the samples irradiated with He^2+^ ions, the main changes in the decrease in hardness values and crack formation resistance are observed for fluences greater than 5 × 10^16^ ions/cm^2^, which evidences the processes of accumulation of radiation damages caused by irradiation and the following implantation of He^2+^ ions. On the other hand, at fluences less than 5 × 10^16^ ions/cm^2^, the changes in cracking resistance and hardness are not more than 1–3% and the hardness value changes less intensively than the cracking resistance.

According to the estimated data, the implantation of He^2+^ ions in the I-PyC layer at fluences of 5 × 10^16^–7 × 10^16^ ion/cm^2^ is not more than 0.3–0.5 at. %. At that level, the basic depth of penetration of He^2+^ ions is not more than 200–300 nm, which leads to the formation of defective areas in this layer, resulting in lower strength and hardness. A further increase in the irradiation fluence leads to a decrease in the cracking resistance and hardness values to 5–12%, which indicates that the accumulation of radiation damage negatively affects the resistance of the near-surface layer to mechanical influences.

For the samples irradiated with Kr^15+^ and Xe^22+^ ions, the significant changes of strength characteristics are observed for irradiation fluences higher than 5 × 10^13^ ion/cm^2^ but, at the maximum irradiation fluence, the decrease in strength characteristics does not exceed 15–20%. Moreover, for Xe^22+^ ions, due to the fact that their initial energy is higher and, consequently, the values of energy losses in the material along the trajectory of motion are higher, the degree of change in strength properties is more pronounced than for Kr^15+^ ions.

In general, the decrease in cracking resistance is caused by the effects of accumulation of radiation damage in the structure of the near-surface layer when the irradiation fluence increases. At the same time, as is known, the increase in irradiation fluence for heavy ions Kr^15+^ and Xe^22+^ above 10^12^–10^13^ ions/cm^2^ leads to the formation of overlapping areas of point defects, with subsequent formation of their conglomerates or complexes, which leads to the appearance of additional deformation contributions in the structure and strengthening of their pressure on the structure. As a result, crystalline and chemical bonds are partially destroyed, leading to embrittlement and destruction of the near-surface damaged layer. 

## 4. Conclusions

The paper presents the results of characterizing the morphological, structural, and mechanical properties of model prototypes of new generation TRISO fuel designed for Generation IV HTGR-type reactors. The methods used to characterize the properties of the studied samples were scanning electron microscopy, atomic force microscopy, energy dispersive analysis, and X-ray diffraction. In the course of the experiments it was determined that the initial samples are spheres consisting of three different layers and a core. According to the data obtained, the structure of the studied particles consists of the upper layer of pyrolytic carbon, the second layer of silicon carbide, a thin layer of porous pyrolytic carbon, and a core of silicon carbide and carbon particles. It was found that, in the initial state, the sphere surface has a developed structure consisting of fine-grained inclusions located on the surface with a high proportion of uniformity. Using the method of X-ray phase analysis, it was found that for the SiC phase the value of integral porosity of the studied samples is 1.61%, with a phase density of 3.134 g/cm^3^. The degree of crystallinity of the ceramic layer of silicon carbide is more than 95%, which indicates a high degree of structural ordering. 

During the studies, it was found that the samples under study have a high degree of resistance to external mechanical influences, which is due to the high strength of the upper layer consisting of pyrolytic carbon.

The results of the radiation resistance of the studied samples showed high resistance of TRISO to radiation damage caused by irradiation with both low-energy He^2+^ ions and high-energy Kr^15+^ and Xe^22+^ ions.

Further research will focus on corrosion and degradation resistance tests, as well as interactions with the gas environment, which will simulate more closely real reactor conditions. Thus, when studying corrosion resistance, the fact that the studied TRISO particles must be placed in a graphite matrix, which also affects the cooling and interaction of particles with the environment, will be taken into account.

## Figures and Tables

**Table 1:** Technical characteristics of TRISO fuel coatings.

Coating Layers	Technical Characteristics
Layer thickness (μm)	
Buffer	95 ± 30
I-PyC	40 ± 8
SiC	35 ± 5
O-PyC	40 ± 6
Layer density (chemical composition) (g/cm^3^)	
Buffer (Pyrolytic carbon)	1.05 + 0.15/−0.05
I-PyC (Pyrolytic carbon)	1.85 ± 0.10
SiC (Silicon carbide)	≥3.19
O-PyC (Pyrolytic carbon)	1.85 ± 0.10

## Data Availability

The data presented in this study are available on request from the corresponding authors.
